# Optimization of the Iron Ore Direct Reduction Process through Multiscale Process Modeling

**DOI:** 10.3390/ma11071094

**Published:** 2018-06-27

**Authors:** Rami Béchara, Hamzeh Hamadeh, Olivier Mirgaux, Fabrice Patisson

**Affiliations:** 1 Institut Jean Lamour, CNRS, Université de Lorraine, 54011 Nancy, France; rami.bechara@univ-lorraine.fr (R.B.); hamzeh.hamadeh@univ-lorraine.fr (H.H.); olivier.mirgaux@univ-lorraine.fr (O.M.); 2 Laboratory of Excellence on Design of Alloy Metals for Low-Mass Structures (DAMAS), Université de Lorraine, 57073 Metz, France

**Keywords:** ironmaking, direct reduction, iron ore, DRI, shaft furnace, mathematical model, simulation, CO_2_ emissions

## Abstract

Iron ore direct reduction is an attractive alternative steelmaking process in the context of greenhouse gas mitigation. To simulate the process and explore possible optimization, we developed a systemic, multiscale process model. The reduction of the iron ore pellets is described using a specific grain model, reflecting the transformations from hematite to iron. The shaft furnace is modeled as a set of interconnected one-dimensional zones into which the principal chemical reactions (3-step reduction, methane reforming, Boudouard and water gas shift) are accounted for with their kinetics. The previous models are finally integrated in a global, plant-scale, model using the Aspen Plus software. The reformer, scrubber, and heat exchanger are included. Results at the shaft furnace scale enlighten the role of the different zones according to the physico-chemical phenomena occurring. At the plant scale, we demonstrate the capabilities of the model to investigate new operating conditions leading to lower CO_2_ emissions.

## 1. Introduction

Steel is one of the key materials of today’s industrial world. Moreover, its production is characterized by high energy consumption along with important carbon dioxide emissions. World steel production amounts to 6% of anthropogenic CO_2_ emissions [1]. Beside the current reference steelmaking route (integrated plant with sinter and coke making, blast furnace, and basic oxygen furnace), less CO_2_ emitting alternative routes are attracting greater interest. Direct reduction (DR) in a shaft furnace followed by electric arc steelmaking thus results in 40 to 60% lower CO_2_ emissions compared with the reference route [2]. Direct reduction converts solid iron ore pellets into so-called direct reduced iron (DRI), using a mixture of CO and H_2_ produced by reforming natural gas. Due to independence from coke and coal imports, sizeable units to meet demand, reduced investment costs, and reduced construction time [3], direct reduction units are becoming more numerous, particularly in countries where natural gas is cheap and abundant. These advantages have spurred increased research activity, namely in modeling and simulation, in order to correctly evaluate these processes and ultimately optimize them. The present work contributes to this effort with the key objective to provide an accurate simulation of the whole DR process, enabling us to propose new avenues for CO_2_ emission mitigation. A distinctive feature of our approach is the multiscale nature of modeling, from, say, 1 μm (grain size) to 1 hm (plant size). We thus developed a single pellet model, a shaft furnace model, and a plant model, using different and complementary modeling strategies. These are presented in the next sections, together with references to previous works.

## 2. Single Pellet Model

**Figure 1:**
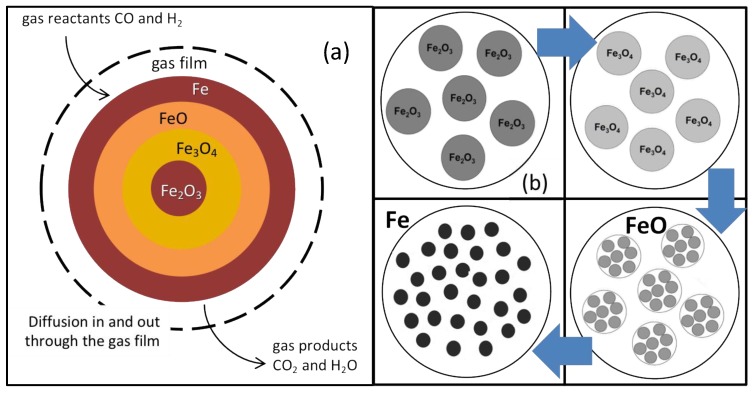
Evolution of pellet structure along with reaction: (**a**) Unreacted Shrinking Core Model; (**b**) Grain Model. The porous structure evolution (**b**) was determined from experimental observations [7].

Iron ore pellets (roughly spherical, typically 7–15 mm diameter) for DR are industrially produced from natural hematite grains (irregular, typically 20 μm). In a shaft furnace, these pellets are chemically reduced at 600–950 °C by both CO and H_2_ in three steps, hematite Fe_2_O_3_ ⟶ magnetite Fe_3_O_4_ ⟶ wustite Fe_0.95_O ⟶ iron Fe, thus involving six gas-solid reactions at the grain scale. Numerous models for gas-solid reactions are available in the literature, from the simplest unreacted shrinking core model to pore models and grain models (Figure 1) [4]. The grain model matches well the grainy structure of the DR pellets; however, it was not used as such in the current study because it requires a numerical solution of the diffusion-reaction equations, which is incompatible for computation time reasons because of its integration in a multi-particle reactor model (next section). Instead, we used an alternate approach, the additive reaction times law, which gives an approximate analytical solution to calculate the reaction rates and can account for mixed regimes [5]. According to experimental observations, we also introduced an evolution of grain and pore structure with the reactions as follows: the hematite grains become covered with small pores after conversion to magnetite and wustite. At the wustite stage, the pores enlarge and the wustite grains break down into smaller particles termed “crystallites”. These subsequently grow (typically from 1 to 10 μm) and join to form the molten-like structure of sponge iron (another name for DRI). These changes of course influence the kinetics of the transformation, mostly via the diffusion resistances. Details of the single pellet model used and of its results are given in [6,7].

## 3. Shaft Furnace Model

### 3.1. Previous Works

**Figure 2:**
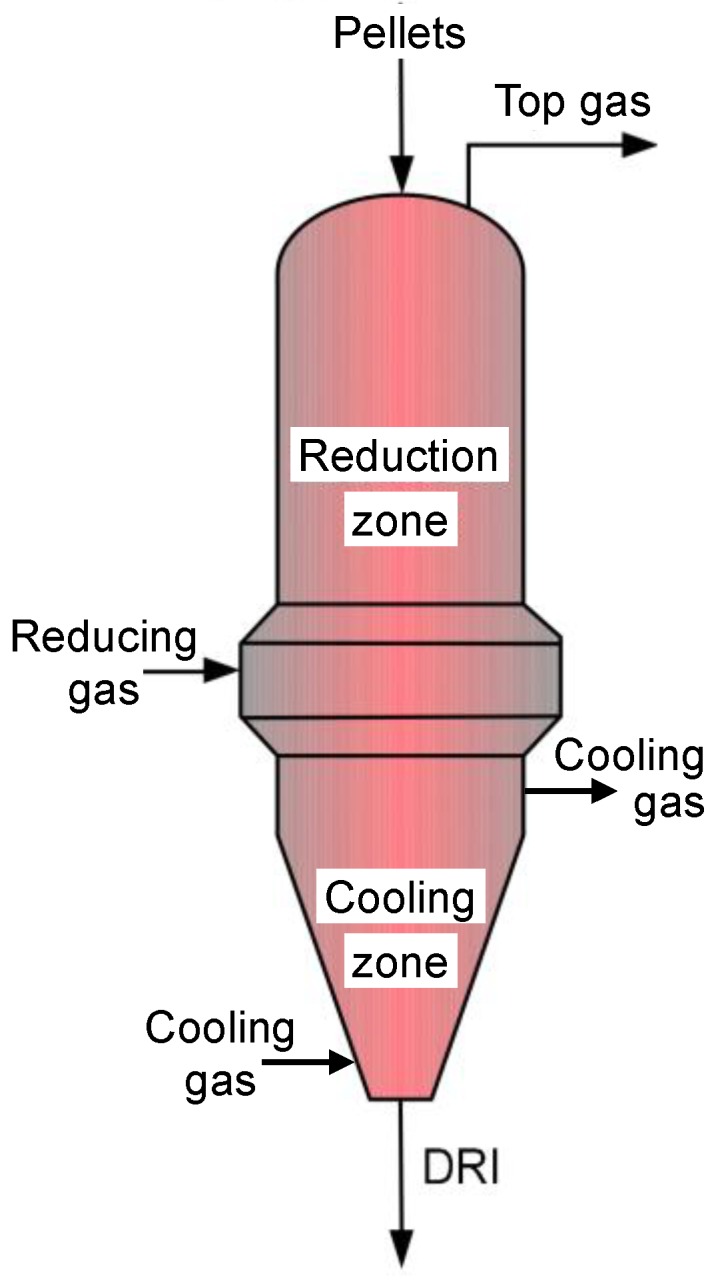
Schematic layout of a direct reduction shaft furnace.

The shaft furnace (Figure 2) is the core of the DR process. Iron ore pellets are charged at the top, descend due to gravity, and encounter an upward counter-flow of gas. The reducing gas (CO and H_2_, plus CH_4_, CO_2_, and H_2_O, at about 950 °C) is injected peripherally at mid-height and exits at the top. In the lower section of the furnace, of conical shape, cold natural gas is injected to cool the iron pellets produced. The upper section (reducing zone and intermediate zone) is cylindrical (typically height 15 m; diameter 5 m). Two processes, MIDREX and HYL-ENERGIRON, share most of the DR market. Their shaft furnaces exhibit some differences (mostly in gas composition and pressure, size, and internal equipment details) but their basic characteristics are similar.

Reflecting the great effort put in the simulation and development of the DR processes, numerous mathematical and numerical models of the shaft furnace were published, differing by the assumptions, the physico-chemical phenomena accounted for, the numerical scheme, the sections of the furnace considered, and the use of the model, etc. The most recent ones [6,7,8,9,10,11,12,13,14,15] are classified in Table 1 and Table 2 and below. The basic description is that of an axisymmetric, porous moving bed reactor with counter-current flow between an ascending gas and a descending solid. The main differences are as follows:

- The number of reduction steps. This is related to the intermediary iron oxides taken into account. Works with one reduction step consider the direct transformation of hematite to iron, those with two steps consider further the presence of wustite, whereas those with three steps consider also magnetite.
- The nature of the inlet gas mixture. Most authors considered an inlet gas consisting of CO and H_2_ only. Two models [13,14] considered the actual mixture, with CO_2_, H_2_O, N_2_, and CH_4_ as additional gas components.
- The number of dimensions included in the geometrical description. The standard is one-dimensional models. However, three works [6,14,15] considered two dimensions: along the height and the radius of the furnace.
- The description of the pellet transformation: shrinking core or grain models.
- The presence of supplementary reactions. The reduction reactions (in one, two or three steps) are always included. Additionally, methane decomposition and steam methane reforming were also taken into account in [10,12,13,14], together with the water gas shift and Boudouard reactions in [14].
- The type of heat transfer. All works included heat convection between solid and gas, as well as the heat of reaction. Heat transfer by conduction and radiation, through a contribution to effective conductivity, was also sometimes considered [7,8,11,14,15].
- Pressure drop was only included in [10,14].
- The presence of the cooling zone was only taken into account in three papers [10,13,14].
- Some models were validated against plant data, others not.

Results that are common to these works are as follows:

- The molar content of H_2_ and CO decreases from the reduction zone bottom to its top whereas that of CO_2_ and H_2_O increases. Inversely, the content of iron oxides decreases from top to bottom with mainly iron exiting from the shaft bottom.
- The solid and gas temperatures are equal along the shaft except in a thin layer at the top near the solid inlet where a great temperature difference exists, the pellets are charged cold. Moreover, both temperatures increase from the shaft top to its bottom.
- Reaction enthalpy is of great importance, namely, the rather endothermic nature of H_2_ reduction reactions vs. the exothermic nature of CO reduction reactions, as well as for the models that include it, the endothermic nature of steam methane reforming.

Key points that can be deduced when comparing differing results indicate that:

- the inclusion of three reduction steps and the grain model better represent the pellet transformation;
- all the components of the gas mixture (excepting N_2_ but including CH_4_) play a role in the transformations;
- two dimensions depict more accurately the reactor internal behavior and the output results; 2D results revealed the presence of two zones: one peripheral where the bustle gas is the reducing gas, and one central where the gas stems from the cooling and transition zones;
- taking supplementary reactions into consideration along with the cooling and transition zones better represents gas phase transformations and can account for carbon deposition.

The present shaft model has been built considering these results and on the basis of the most detailed (2D, 3 zones, 10 reactions–named REDUCTOR) shaft model [6,14]. However, the goal here is to simulate the whole DR plant, thus it was not practicable to incorporate such a comprehensive, 2D, CFD-type model in the plant simulation software. We selected Aspen Plus, a commercial simulation tool widely used in the chemical industry, as the software. Processes like the steelmaking blast furnace and the reverse chemical looping process are examples of processes somewhat close to the DR process that have been modeled with Aspen Plus. Thus, we had to build an Aspen Plus model of the DR shaft derived from REDUCTOR.

### 3.2. Aspen Plus Shaft Model

**Figure 3:**
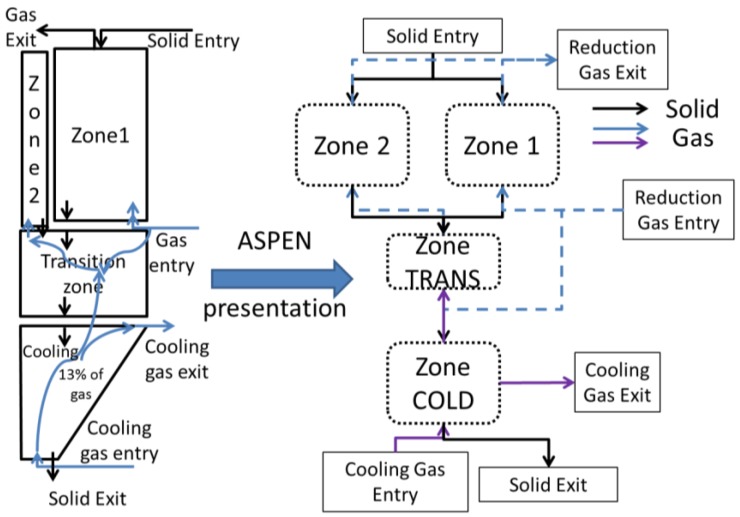
Sectioning of the shaft furnace for the Aspen Plus model.

The results given in [14] show that, in addition to the initial 3-stage division, the reduction zone can be sub-divided into two zones. In the first one, Zone 1, the widest, peripheral zone, the oxides are almost completely reduced at the bottom of the zone. The inlet gas in this zone is the major fraction of the bustle reducing gas plus a part of the gas coming upward from the transition zone. The inlet solid is the major fraction of the oxide pellets charged. The second zone, Zone 2, is a narrow central zone in which only partial metallization occurs. Its inlet gas comes from the transition zone, whereas its inlet solid is a fraction of the oxide pellets. Figure 3 depicts this partitioning and also shows its translation into an Aspen Plus based configuration.

**Figure 4:**
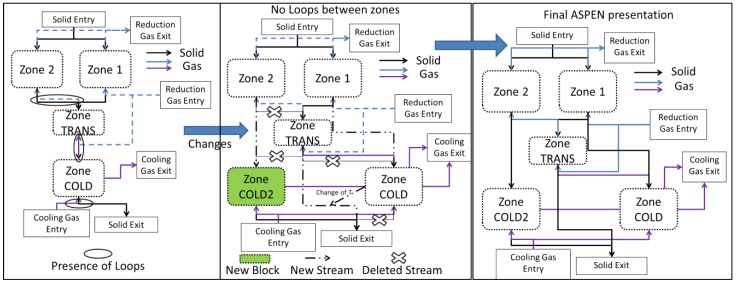
Restructuring of Aspen Plus configuration of the shaft furnace to avoid loops.

Nevertheless, due to the countercurrent of the solid and gas flows, this description implies using several loops for the numerical solution; e.g., the gas coming from the transition zone influences the solid in the central zone, which in turn influences the transition zone gas. Likewise, the solid from the transition zone influences the gas from the cooling zone, which in turn influences the solid. These loops make it difficult and time-consuming to simulate the process. An effort was thus made to simplify the model. The flow rate of the solid in the central zone being considerably smaller than that in the peripheral zone, the former was not considered as an input to the transition zone, thus cancelling the first loop. Moreover, the transition zone had every little impact on the temperature of the descending solid, thus the link between the descending transition solid and the cooling zone was removed. In place, a fictitious solid stream equal to that leaving the peripheral zone was used as an input. The resulting temperature was thus imposed on the solid leaving the transition zone. This solid was considered as the ultimate output of the peripheral zone. Lastly, an additional cooling zone was added for the central zone. Attention was made to avoid any loops between the two sections. Considering this, Figure 4 highlights the restructuring of the Aspen Plus configuration to avoid the aforementioned loops. Herein, the cross signs emphasize deleted streams, which were responsible for loops, whereas the dotted lines indicate new streams. As a result of this restructuring, a new cooling zone (COLD2) was added and the resulting temperature from the cold section was returned to the exit of the transition zone. Of course, these changes, made for the sake of simplification, do not alter the overall mass and heat flowrates and balances.

This representation however poses the problem of finding the adequate split for the streams between the various sections. We identified three critical splits. The first is at the reduction gas entry level, the bustle gas being split between the transition zone and Zone 1. The second is at the top solid inlet, the entering solid being split between Zone 1 and Zone 2. The third split is at the cooling gas stream that goes up to the transition zone. The first split was set to a predefined value. The second split was calculated considering that the ratios of the inlet solid flow to the input gas flow for each zone are equal. The third split was set to the constant value of 0.13 as given in [6].

This representation, set and done, is not yet sufficient to directly calculate the final conversion and temperatures. Especially for the transition and reduction zones, Aspen Plus does not offer any built-in model that allows their calculation in a way that could correctly depict the reaction kinetics and the variations of the different variables along the height of the shaft. An external calculator, written in Fortran and based on REDUCTOR’s equations, was therefore used to compute these values, which were later rendered to the corresponding Aspen Plus blocks. Conversely, for the cooling section, a simple Aspen Plus heat exchanger could be used. The principle of the calculation, namely in the reduction and transition zones, and the list of reactions are given in Appendix A.

### 3.3. Results from the Aspen Plus Shaft Model

Two case studies were considered in this work, based on real industrial data. The first case corresponds to the Contrecoeur plant, located near Montreal, Canada, and currently operated by ArcelorMittal. It was constructed in the late 1970’s and is a MIDREX series 750 module. It is characterized by a rather cold input solid and a high content of C in the input gas. The second case relates to the Gilmore plant, built near Portland, Oregon, USA. Now decommissioned, it was the first operating MIDREX plant. It is one of the most referenced plants in literature, a MiniMod module with a CDRI production of 26.4 tons/h. The input operating conditions for both plants are given in Appendix B. These are of quite different capacities, with different reducing gas compositions and different pellet diameters, and thus represent a good test for validating a model.

Table 3 compares the results obtained in the model for the outlet gas and solid streams with those provided in literature for the studied cases. The difference for each parameter was calculated as well as the total error. As can be seen, the absolute error for the Contrecoeur case is equal to 6%, whereas that for Gilmore is 4%. The biggest differences pertain to methane and nitrogen composition as well temperature. Other differences include hydrogen and water compositions in the Gilmore case, and the carbon dioxide composition in the Contrecoeur case. These differences can be related to the formulas chosen from the literature for the gas phase reaction rates. For example, methane decomposition and Boudouard reactions seem to be somewhat underestimated in Contrecoeur and overestimated in Gilmore. These values could only be further consolidated through the realization of up-to-date experiments to correctly characterize these reactions. Nonetheless, the results seem to be globally satisfactory, especially if they are compared with other model results [10,14]. The related differences are comparable although the present model is of a different type and simpler than the two CFD-type models.

**Figure 5:**
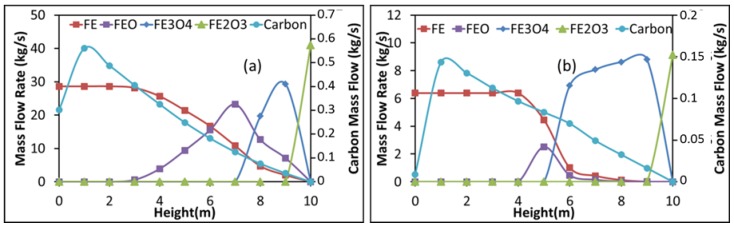
Evolution of the solid component flow rates with shaft height in Zone 1, (**a**) Contrecoeur; (**b**) Gilmore.

Figure 5 shows the evolution of the solid component flow rates with shaft height in the first reduction zone for the Contrecoeur (a) and Gilmore (b) cases. Height zero corresponds to that of the bustle gas inlet. As can be seen, hematite disappears very rapidly near the solid inlet in both cases. Magnetite on the other hand has a different behavior; it disappears after 2.6 m in the Contrecoeur case, against 4.6 m in the Gilmore case, with more of this oxide formed in the latter case. Wustite on the other hand disappears rapidly in the Gilmore case, its presence window being only 2 m, against about 7 m in the Contrecoeur case. Finally, as expected, both cases give a 100% conversion of iron oxide to pure iron in this Zone 1. Figure 5 also shows the evolution of the carbon deposited in the pellets, which continuously increases from solid entrance before dropping near gas entrance. Exit solid has some carbon content in the Contrecoeur case, whereas the carbon content drops to zero in the Gilmore case. This is related to the inversion of the Boudouard equilibrium (C + CO_2_ ⇌ 2CO), which is favorable to carbon deposition at lower temperatures and lower CO_2_ content.

**Figure 6:**
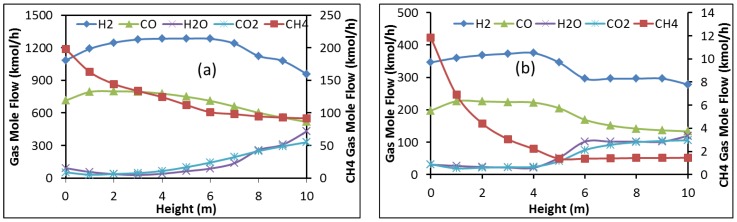
Evolution of the gas component flow rates with shaft height in Zone 1, (**a**) Contrecoeur; (**b**) Gilmore.

These profiles can be related to the gas phase reactions, which are presented for both cases in Figure 6. It can be seen that methane, water, and carbon dioxide flow rates decrease above the gas entrance, whereas those of hydrogen and carbon monoxide increase. This is the opposite in the upper half of the shaft, except for methane, which sees its flow rate reach equilibrium. This inversion can be explained by the preponderance of the iron oxide reduction reactions, as well as the inverse Boudouard reaction (2CO ⇌ C + CO_2_) as evidenced by the carbon production in Figure 5. The decrease in methane can be related mainly to the steam reforming (CH_4_ + H_2_O ⇌ CO + 3H_2_) with carbon deposition from methane (CH_4_ ⇌ C + 2H_2_) having a rather negligible impact. This little impact is emphasized by the decline in carbon flow rate near the gas inlet, which is due to the direct Boudouard reaction.

**Figure 7:**
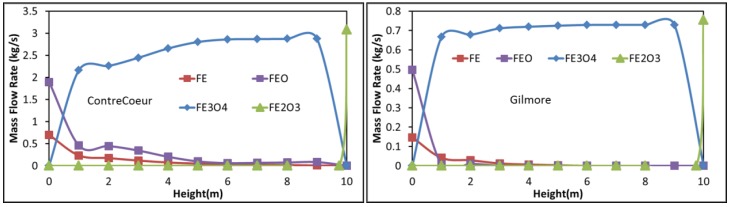
Evolution of the solid component flow rates with shaft height in Zone 2, (**a**) Contrecoeur; (**b**) Gilmore.

Among the other calculated results (temperature, reaction rates, and values for the other zones), we selected for presentation and comparison the evolution of the iron compounds flow rates with shaft height in Zone 2 (Figure 7). The situation differs from that of Zone 1. Whereas hematite is readily reduced into magnetite as previously reported, magnetite remains present over most of the height, being slowly reduced in the Contrecoeur case and almost not changing in the Gilmore case until the gas inlet. Conversion to wustite and iron only occurs at the zone bottom, with 60% wustite remaining in the solid at the exit. This situation results from a lower temperature and less CO and H_2_ for the reduction in Zone 2 compared with Zone 1. The other reactions, involving methane, carbon monoxide and carbon dioxide, hardly take place in Zone 2. These results emphasize the impact this second zone has on the low exit gas temperature and on the average metallization degree.

The importance of the transition zone should also be noted. The main reaction in this zone is methane decomposition $\left({\mathrm{CH}}_{4}\to C+2H_{2}\right)$. Carbon is herein deposited on produced iron, leading to the final carbon content of the DRI. This carbon comes in addition to the carbon possibly deposited via the inverse Boudouard reaction in Zone 1 (Figure 5). At the rather low temperature of the transition zone, no iron oxide reduction by solid carbon occurs. The hydrogen produced by the methane decomposition reaction is sent to the second zone, contributing to the final metallization degree. Moreover, the gas-solid temperature difference is reversed in this zone, the descending solid being hotter than the ascending gas.

## 4. Plant Model

**Figure 8:**
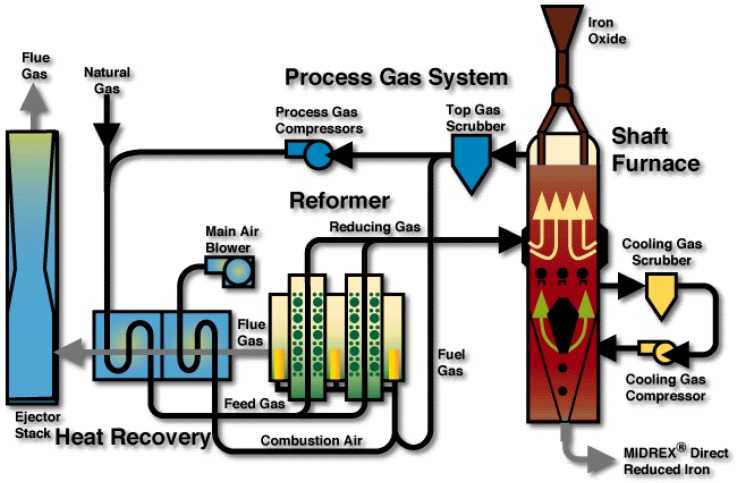
MIDREX direct reduction process [17].

The next scale is that of the whole DR plant, a schematic diagram of which is given in Figure 8 (case of a MIDREX-type plant). The main units around the shaft furnace are: the reformer, the scrubber, and the heat exchanger. Downstream from the shaft furnace the top gas is scrubbed and divided into two streams. The first stream loops in the system. It is mixed with natural gas, reheated, and sent to the catalytic tubes of the reformer to produce the reducing gas. The second one (plus possibly some extra natural gas) is burnt with air to provide the reformer with heat. The hot flue gas is used in an exchanger to heat both gas streams entering the reformer. The MIDREX reformer is a kind of ‘dry’ reformer where the primary reaction is CH_4_ + CO_2_ ⟶ 2H_2_ + 2CO. In other DR processes, methane is rather reformed by H_2_O (HYL), the reformer is smaller or missing (HYL-ZR) or the reducing gas is produced from other fuels (ENERGIRON). This variety was a further incentive to develop the present DR plant model using Aspen Plus, since we expect to use it for process optimization, including modifying parts of the configuration. Contrary to the shaft furnace, the plant scale was scarcely investigated through modeling. Only two authors studied the interaction between the reformer and the shaft furnace, using mathematical models for each unit [6,16].

### 4.1. Aspen Plus Plant Model

**Figure 9:**
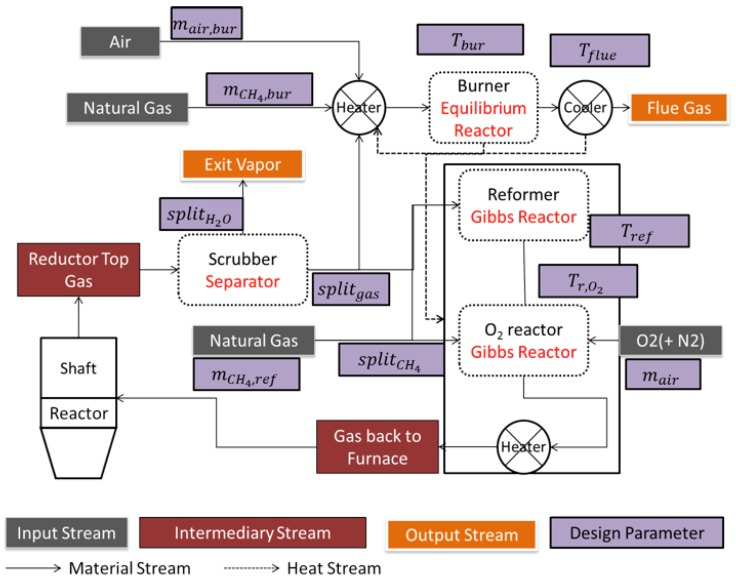
Whole plant Aspen Plus model, with gas recirculation and key design variables.

Figure 9 illustrates the corresponding Aspen Plus model. Starting from the shaft furnace top gas exit the scrubber is the first step, in which part of the water vapor is separated from the rest of the stream. The adopted Aspen sub-model is a splitter, in which the separated H_2_O fraction is defined ($split_{H_{2}O}$). The remaining off-gas going is then split in two streams: one ($split_{gas}$) going to the reformer and the other to the burner. On the other hand, reformer natural gas is also split in two fractions, one sent prior to the reformer ($split_{CH_{4}}$) and the other after it. The reformer was modeled using the built-in Aspen Plus ‘Gibbs reactor’. This reactor does not consider kinetics, but calculates the product composition minimizing its Gibbs energy; i.e., at equilibrium. This is a simplification often used in the literature [18,19]. The key design variable is *T_ref_*, the reforming temperature. The gas stream exiting the reformer is later mixed with natural gas and air prior to being sent back to the shaft furnace. A prior step was added in the form of a Gibbs reactor where O_2_ injection and burning is realized. Herein, the reactor temperature ($T_{r,O_{2}})$ is the key design variable. The combustion system on the other hand has as inlets the remaining off-gas, natural gas, and combustion air. These streams are heated to the combustion temperature, burnt in the burner producing hot flue gases later cooled prior to discharge. The burner was also modeled as a stoichiometric reactor, employing gas combustion reactions, with its temperature ($T_{bur})$ also as a key design variable. The burner’s goal is to provide energy for the reforming system. This step is then followed by flue gas cooling in order to recover energy, namely for pre-heating purposes. The key flue gas cooling variable is ($T_{flue}$).

The principle used for modeling the plant in Aspen Plus was to make use of Aspen Plus Design Specs. A Design Spec is a condition that the Aspen Plus solver will make satisfied by acting on a ‘controlled variable’. In this case, on the one hand, reformer variables were controlled so that the gas exiting the reformer is identical to the bustle gas, leading to a closed looped process. On the other hand, burner variables were controlled in order to guarantee an adequate heat balance in the process. The design specs refer to the equality of flow rates for hydrogen, water vapor, carbon monoxide, carbon dioxide, methane, and nitrogen between the bustle-gas and the exit gas respectively. The controlled variables are: $split_{gas}$, $split_{H_{2}O}$, $T_{ref}$, $T_{r,O_{2}}$, $n_{CH_{4},ref}$, $n_{air}$, $split_{CH_{4}}$, $n_{CH_{4},bur}$, $T_{flue}$ and $n_{air,bur}$.

### 4.2. Results from the Aspen Plus Plant Model

Table 4 lists the various design specifications as well as the corresponding controlled variables, and the resulting values. The obtained values were compared to available data in the case of Contrecoeur; no such data were available for Gilmore. As it can be seen, the values do not differ in the first case except for $split_{CH_{4}}$. Indeed, this is not really a discrepancy given that the same amount of methane is reformed. According to plant data, 92% of the methane is sent to the reformer where 46% of it is reformed, the yield being limited by kinetics. Conversely, the reformer modeled as a Gibbs reactor gives an almost complete reforming and thus only 43% of the methane is needed in the reformer, the rest being by-passed. Another approach would have been to adopt a 1D model of the reformer with kinetics, like [6,16]. Downstream of the reformer, the gas temperature is adjusted using a small air injection.

Results in the Gilmore case resemble the Contrecoeur case for $T_{ref}$ and $n_{CH_{4}.bur}$. The first is related to equilibrium whereas the second showcases the lack of need for excess fuel. Both processes however differ for the remaining variables. In the Gilmore case, $split_{gas}$ has a higher value leading to greater off-gas recycling. This may indicate the need for greater CO_2_ reforming as well as the need to conserve unreacted CO-H_2_ reductants present in the off-gas. The smaller $split_{H_{2}O}$ value can be understood in light of a greater H_2_O reforming in the connected reformer. The smaller amount of natural gas sent to the reformer is related to the smaller gas flow rate entering the shaft furnace. The equivalent input air flow rate can be related to the greater amount of nitrogen present in the gas entering the furnace. Higher $split_{CH_{4}}$ values can be understood in light of the equilibrium that is reached in the reformer but also a smaller methane fraction in the gas stream entering the shaft furnace. The greater amount of input process air $n_{air,ref}$ required in the burner is related to the greater N_2_ flow rate in the bustle gas.

### 4.3. Process Visualization

**Figure 10:**
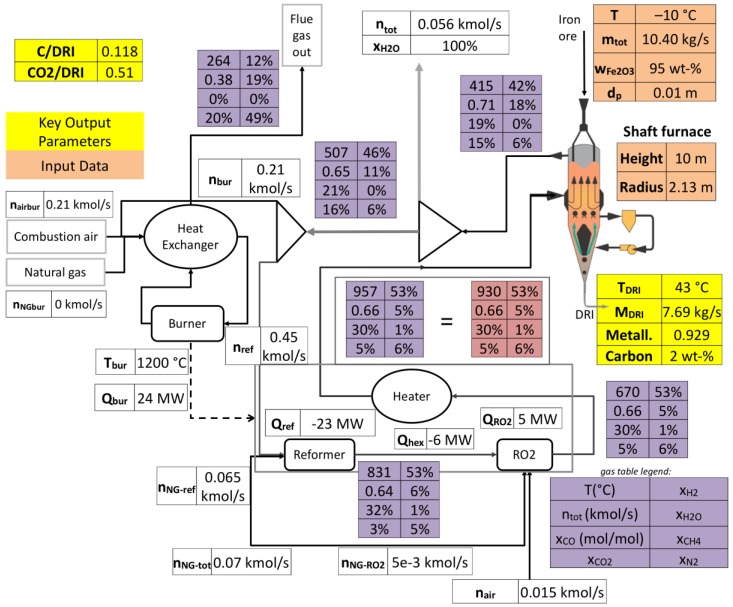
Simulated process flow diagram (Gilmore case).

The adopted process model and simulation tool further enable us to create a mimic board of the plant. Figure 10 highlights the flow diagrams of this process in the Gilmore case. As can be seen, all process units are shown, the shaft reactor, scrubber, reformer, oxygen injection, burner, and air preheater. Moreover, all streams to and from these units are highlighted along with their composition. The figure puts a great emphasis on the gas loop, by providing temperature and composition changes along the operations; e.g., the reduction in H_2_, CO and CH_4_ content in the shaft reactor and their recovery in the reforming system. Moreover, it can be seen that the burner provides the reformer system with heat by burning part of the scrubbed off-gas, with little need for input methane. Certain key parameters are shown in yellow, like the mass flow rate of DRI, its metallization and carbon content, together with the ratio of equivalent carbon present in the flue gas to the quantity of DRI produced (C/DRI, here equal to 0.119) as well as the same ratio but for equivalent CO_2_ (CO_2_/DRI). In fine, this visualization makes it easier to compare simulations to plant data or to compare simulation runs between each other.

### 4.4. Process Optimization

Having modeled the shaft reactor as well as the gas loop system, various process optimization schemes can be considered. Our aim in this paper was to address the reduction in CO_2_ emissions. Keeping the same plant configuration (MIDREX-type), the chosen means was the modification of the inlet reducing gas composition. According to the literature, the two important ratios are $H_{2}/\mathrm{CO}$ and $\left(H_{2}+\mathrm{CO})/(H_{2}O+{\mathrm{CO}}_{2}\right)$; i.e., the hydrogen-to-carbon monoxide ratio and the reducing power of the reducing gas. The initial values for these parameters were respectively equal to 1.5 and 12 for Contrecoeur and 1.75 and 8.73 for Gilmore. Optimal ratios were recorded close to 1 and 12 for both parameters respectively [9,20]. Considering this, optimization runs are provided hereafter for Gilmore. The initial value for the carbon-to-DRI ratio was 0.119, a figure also in line with the literature, where the range of 0.105–0.120 was reported [2].

Table 5 highlights the optimization runs carried out by decreasing $H_{2}/\mathrm{CO}$ from 1.75 to 1.05 and increasing $\left(H_{2}+\mathrm{CO})/(H_{2}O+{\mathrm{CO}}_{2}\right)$ from 8 to 16. As can be seen, the carbon-to-DRI ratio decreases by as much as 12% between the original case and the optimal case. This case corresponded to a $H_{2}/\mathrm{CO}$ ratio of 1.23 and $\left(H_{2}+\mathrm{CO})/(H_{2}O+{\mathrm{CO}}_{2}\right)$ of 12. Attempts to further decrease $H_{2}/\mathrm{CO}$ led to model divergence, the reformer not being able to produce the desired bustle gas composition. Increasing the $\left(H_{2}+\mathrm{CO})/(H_{2}O+{\mathrm{CO}}_{2}\right)$ ratio over 12 did not further reduce the carbon-to-DRI ratio.

These results illustrate the potential of the tool. The key differences between the optimized and the original operations pertain to greater $split_{H_{2}O}$ (0.72 vs. 0.44) and $split_{gas}$ ratios (0.75 vs. 0.64). More $H_{2}O$ is thus withdrawn in the scrubber, whereas greater gas recycling occurs. This can be translated in a greater importance for CO_2_ reforming and a greater conservation of the reducing gases. This conservation further leads to a smaller requirement of reformer natural gas (64 mol/s vs. 71 mol/s). Also, a smaller energy demand occurs in the reformer (22 MW vs. 24 MW). It should be further noted that these results were obtained without little (but positive) changes in metallization degree (93.3% for the optimal case vs. 93% for the original case) and carbon content (2% vs. 1.8%).

Calculations were also performed for the Contrecoeur case. The best carbon-to-DRI ratio was 0.099 kg/kg (a 17.5% reduction from the original case 0.12 kg/kg), obtained using $H_{2}/\mathrm{CO}$ and $\left(H_{2}+\mathrm{CO})/(H_{2}O+{\mathrm{CO}}_{2}\right)$ ratios of 1.39 and 12.5, respectively. These values are in line but somewhat different from those of the Gilmore case, due to the content in other gas components of the bustle gas.

## 5. Discussion

The obtained results were already given a first interpretation in the previous sections. Hereafter, we focus on the context and significance of the present work. The developed process model differs from previously published works by its multi-scale nature, from grains (μm) to the shaft furnace (hm), and ultimately to integrated plant simulation (Aspen Plus).

The core of the DR process is the reduction shaft furnace. The corresponding Aspen Plus model was made sufficiently sophisticated to reproduce the main results from the more complex, CFD-type models of this reactor, but sufficiently light enough to be integrated in a whole plant flowsheet model. Thus, based on the recent results of [14], which proposed a virtual division of the shaft into zones distinguished according to the physico-chemical and thermal phenomena occurring, the shaft furnace was modeled as a set of interconnected zones. The ones in the reduction and intermediate sections were discretized in horizontal slices. This description, intermediate between 1D and 2D, enables us to retain the advantages of describing the axial variations of the calculated variables and of a short computation time (typically 3 h on a standard PC). The results from this model agree well with both available industrial data and results from previous models. Moreover, the two cases simulated corresponding to plants of different operating conditions and of quite different throughput, this demonstrates the adaptability and the robustness of the model.

The overall Aspen Plus model of a DR plant includes models for the principal units: the shaft furnace, the natural gas reformer, the scrubber, and the main heat exchanger. To the best of our knowledge, this is the first published work on a systems model of the whole DR plant using process simulation software. The decisive interest of such an approach is that the recycling gas can be accounted for. In MIDREX-type DR plants, most of the top gas exiting the shaft furnace is recycled, being first sent to the reformer then fed as the reducing gas at the gas inlet of the shaft furnace. Using the overall model, it becomes possible to study the interactions between the respective performances of the reformer and of the shaft furnace.

As a test seeking to mitigate the CO_2_ emissions from the plant, the reducing gas composition at the shaft gas inlet was varied. A series of simulations show that CO_2_ emissions and natural gas consumption could be reduced tuning the ratios $H_{2}/\mathrm{CO}$ and $\left(H_{2}+\mathrm{CO})/(H_{2}O+{\mathrm{CO}}_{2}\right)$ at 1.23 and 12, respectively. The C-to-DRI ratio can be lowered from 0.119 to 0.105 kg/kg, a 12% reduction.

However, this optimization by hand is a first piece of work. The next stage would be to use a mathematical optimizer for the same purpose. The overall model could also be used to test different plant configurations. A variety of DR plants (MIDREX-type of different sizes, HYL-type of different sizes, HYL-ZR, ENERGIRON with different reducing gas sources) were designed and built. This means that further configurations could be designed to optimize criteria like the CO_2_ emission, but also energy consumption, operating costs, etc. The present model can give useful answers.

## 6. Conclusions

In conclusion, this paper showcases a systemic, multiscale process model developed for the simulation, visualization, and further optimization of a gas-based DR iron reduction plant. A multi-scale approach was adopted, going from grain-size to model the iron pellet and its transformation, to reactor-size where reaction kinetics and heat and mass exchanges were simulated, and ultimately plant-scale where the gas recycling loop was modeled to obtain a closed process, and the subsequent carbon emissions were calculated for two cases. Simulation results were found in good agreement with industrial data. Moreover, hand optimization tests provided a 12% decrease in carbon emissions. Future works will address computer-based optimization along with the testing of novel process configurations.

## Figures and Tables

**Table 1:** Characteristics of DR shaft furnace mathematical models (1).

Reference	Reduction Steps	Gas Mixture	Dimen-sions	Geometric Model	Supplementary Reactions
Rahimi and Niksiar [8]	3	H_2_-CO	1	Grain Model	no
Nouri et al. [9]	1	H_2_-CO-H_2_O-CO_2_-N_2_-CH_4_	1	Grain Model	no
Shams and Moazeni [10]	2	H_2_-CO-H_2_O-CO_2_-N_2_-CH_4_	1	Unreacted Shrinking Core Model	methane decomposition + reforming
Palacios et al. [11]	1	H_2_-CO	1		no, but heat of reaction considered
Bayu and Alamsari [12] Alamsari et al. [13]	3	H_2_-CO-H_2_O-CO_2_-N_2_-CH_4_	1		methane reforming and water gas shift
Ranzani da Costa et al. [7]	3	H_2_	2	Grain Model (hematite & magnetite), Crystallite Model (Wustite)	no
Hamadeh et al. [6,14]	3	H_2_-CO-H_2_O-CO_2_-N_2_-CH_4_	2		methane decomposition, reforming, water gas shift, Boudouard
Ghadi et al. [15]	3	H_2_-H_2_O	2	Unreacted Shrinking Core Model	no

**Table 2:** Characteristics of DR shaft furnace mathematical models (2).

Reference	Heat Contribution	Presence of Cooling & Transition Zones	Pressure Drop	Validation against Industrial Data
Rahimi and Niksiar [8]	reaction, convection, diffusion	no	no	no
Nouri et al. [9]	reaction, convection	no	no	yes
Shams and Moazeni [10]		yes	yes	yes
Palacios et al. [11]	reaction, convection, diffusion	no	no	no
Bayu and Alamsari [12] Alamsari et al. [13]	reaction, convection	yes	no	yes
Ranzani da Costa et al. [7]	reaction, convection, diffusion	no	no	no
Hamadeh et al. [6,14]		yes	yes	yes
Ghadi et al. [15]		no	no	yes

**Table 3:** Simulation of a shaft furnace. Comparison of the results with industrial data and other model results.

Parameter		Contrecoeur			Gilmore			
		Plant Data	Present Model	Reductor Model [14]	Plant Data	Present Model	Reductor Model [14]	Shams Model [10]
Top gas	CO (vol %)	19.58	18.88	19.89	18.90	19.00	20.87	19.44
	CO_2_ (vol %)	17.09	15.60	14.69	14.30	14.60	13.13	17.54
	H_2_O (vol %)	19.03	19.50	19.52	21.20	17.60	20.61	18.04
	H_2_ (vol %)	40.28	40.20	40.41	37.00	42.20	37.70	37.96
	N_2_ (vol %)	1.02	1.63	1.55	7.67	6.00	8.60	7.02
	CH_4_ (vol %)	2.95	4.19	3.91	-	-	-	-
	Temperature (°C)	285	312	293	n.a.	417	285	474
Bottom solid	Fe_2_O_3_/Fe_tot_ (wt %)	0	0	0	0	0	0	0
	Fe_3_O_4_/Fe_tot_ (wt %)	0	0	0	0	0	0	0
	FeO/Fe_tot_ (wt %)	6.20	6.1	6.00	7.00	7.00	4.70	10.00
	Fe/Fe_tot_ (wt %)	93.80	93.90	94.00	93.00	93.00	95.30	90.00
	C (wt %)	2.00	2.30	2.20	2.00	1.80	0.91	1.42
	Average absolute difference with plant data (%)	-	6.0	4.4	-	4.0	6.9	6.5

**Table 4:** Design specifications, controlled variables and corresponding values.

Equality to Respect	Controlled Variable	Value (Contrecoeur)	Data (Contrecoeur)	Value (Gilmore)
$n_{H_{2}.ref_{out}}=n_{H_{2}.bustle}$	$split_{gas}$	0.64	0.65	0.68
$n_{H_{2}O.ref_{out}}=n_{H_{2}O.bustle}$	$split_{H_{2}O}$	0.62	0.60	0.44
$n_{CO.ref_{out}}=n_{CO.bustle}$	$T_{ref}$	837 °C	-	830 °C
$n_{CO_{2}.ref_{out}}=n_{CO_{2}.bustle}$	$T_{r.O_{2}}$	860 °C	-	670 °C
$n_{CH_{4}.ref_{out}}=n_{CH_{4}.bustle}$	$n_{CH_{4}.ref}$	338 mol/s	380 mol/s	71 mol/s
$n_{N_{2}.ref_{out}}=n_{N_{2}.bustle}$	$n_{air,ref}$	11.6 mol/s	-	14.9 mol/s
$n_{C.ref_{out}}=0$	$split_{CH_{4}}$	0.434	0.92	0.92
$Q_{comb}=Q_{ref}$	$n_{CH_{4}.bur}$	0 mol/s	-	8 mol/s
$Q_{flue}=Q_{heat}$	$T_{flue}$	295 °C	-	345 °C
$n_{O_{2}.flue}=0$	$n_{air.bur}$	871 mol/s	-	216 mol/s

**Table 5:** Optimization runs in the Gilmore case.

Parameter	Original	Opti-1	Opti-2	Opti-3	Opti-4	Opti-5	Opti-6
$\frac{H_{2}}{\mathrm{CO}}$	1.75	1.57	1.5	1.35	**1.23**	1.05	1.23
$\frac{H_{2}+\mathrm{CO}}{H_{2}O+{\mathrm{CO}}_{2}}$	8	12	12	12	**12**	12	16
$\frac{C}{\mathrm{DRI}}$ (kg/kg)	0.119	0.119	0.113	0.109	**0.105**	Diverged	0.106
Metallization (%)	93%	93.3%	93.3%	93.3%	93.3%		93%
$x_{c}\left(\mathrm{wt}\;\%\right)$	1.8%	2%	2%	2%	2%		2%

**Figure A1:**
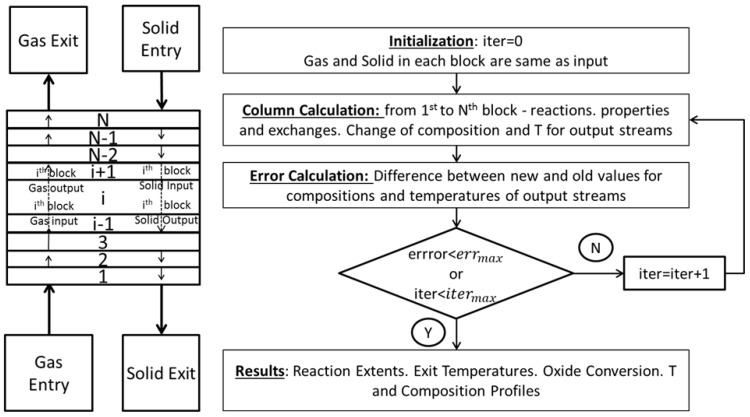
Resolution scheme for the reduction and transition zones.

**Figure A2:**
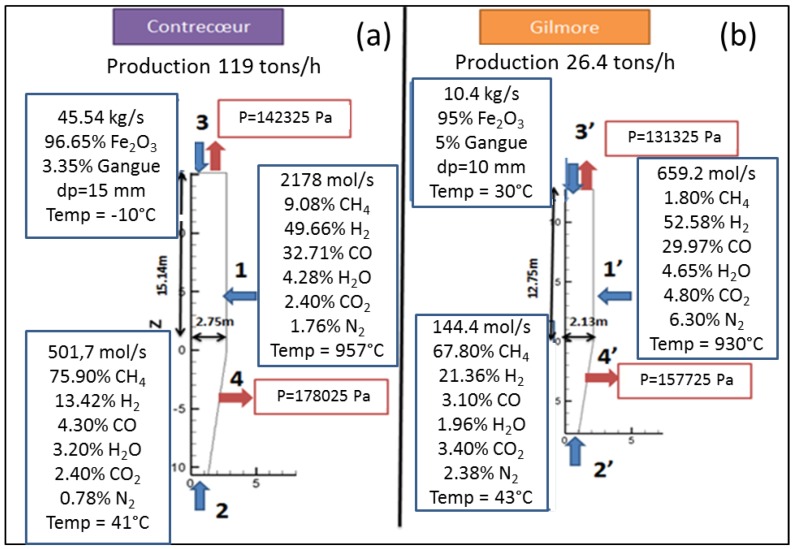
Input conditions of Contrecoeur (**a**) and Gilmore (**b**) plants.

## Appendix A

The calculation scheme for the reduction and transition zones is explained hereafter. A 1D finite difference method was employed. The reactor was partitioned into (4000) horizontal slices of constant surface and equal height (Figure A1).

The calculation loop can be broken down as follows. All gas and solid streams are initialized as equal to the input gas and solid streams respectively. Calculation then starts from the first slice up to the last. Reaction and heat and mass exchanges occur between the ascending gas (i) and the descending solid (i + 1) entering the slice. This exchange impacts the exiting ascending gas (i + 1) and descending solid (i). The difference between the newly calculated values and the previous values is then recorded for each stage. Once the calculation is over, the overall relative error is calculated as the sum of the difference between the old and new value divided by the sum of the total values for each of the following variables: molar flow rate for gas and solid components as well as the temperatures for gas and solid phases. The iteration then continues until either this error is lower than a certain tolerance (10^−4^) or the number of iterations is superior to a maximum number of iterations (7000). Once the calculation is over, results are returned to the Aspen Simulation. These include: the reaction extents, the exit solid and gas temperatures, as well as the temperature and composition profiles for both gas and solid streams along the shaft.

Table A1 provides the list of all the reactions considered in the reduction and transition zones. The first set of equations relate to iron oxide reduction: ${\mathrm{Fe}}_{2}O_{3}\to {\mathrm{Fe}}_{3}O_{4}\to {\mathrm{Fe}}_{0.95}O\to \mathrm{Fe}$, with CO and $H_{2}$ as reduction agents. Their reaction rates are controlled by various resistances (inter-granular, intra-granular, inter-crystallite, intra-crystallite, and chemical) summed together courtesy of the additive reaction times law that considers that the different matter transportation steps occur in series. The rest of reactions are the methane steam reforming, water gas shift, methane decomposition and Boudouard. The kinetic rates are calculated based on stoichiometric factors with a possibility for reverse reactions. The expressions for the kinetic constants are based on literature [17,21,22,23], with the role of iron as a catalyst for methane reactions.

**Table A1:** Considered reactions.

Reaction Number	Name	Formula
Reaction 1	Hematite $H_{2}$ reduction	$3{\;\mathrm{Fe}}_{2}O_{3}+H_{2}\;\to 2{\;\mathrm{Fe}}_{3}O_{4}+H_{2}O$
Reaction 2	Hematite CO reduction	$3{\;\mathrm{Fe}}_{2}O_{3}+\mathrm{CO}\;\to 2{\;\mathrm{Fe}}_{3}O_{4}+{\mathrm{CO}}_{2}$
Reaction 3	Magnetite $H_{2}$ reduction	$1.19{\;\mathrm{Fe}}_{3}O_{4}+H_{2}\;\to 3.75{\;\mathrm{Fe}}_{0.95}O+H_{2}O$
Reaction 4	Magnetite CO reduction	$1.19{\;\mathrm{Fe}}_{3}O_{4}+\mathrm{CO}\to 3.75{\;\mathrm{Fe}}_{0.95}O+{\mathrm{CO}}_{2}$
Reaction 5	Wustite $H_{2}$ reduction	${\mathrm{Fe}}_{0.95}O+H_{2}\;\to 0.95\;\mathrm{Fe}+H_{2}O$
Reaction 6	Wustite CO reduction	${\mathrm{Fe}}_{0.95}O+\mathrm{CO}\;\to 0.95\;\mathrm{Fe}+{\mathrm{CO}}_{2}$
Reaction 7	Steam reforming	${\mathrm{CH}}_{4}+H_{2}O\to \mathrm{CO}+3{\;H}_{2}$
Reaction 8	Water gas shift	$\mathrm{CO}+H_{2}O↔{\mathrm{CO}}_{2}+H_{2}$
Reaction 9	Methane decomposition	${\mathrm{CH}}_{4}\to C+2{\;H}_{2}$
Reaction 10	Boudouard (inverse)	$2\;\mathrm{CO}↔C+{\;\mathrm{CO}}_{2}$

Reaction heat is calculated as the sum of each equation’s heat of reaction and is attributed to the solid phase. Moreover, solid and gas phases transport heat by convection and conduction, and exchange heat through convective transfer.
